# Integrating Artificial Intelligence (AI) Chatbots for Depression Management: A New Frontier in Primary Care

**DOI:** 10.7759/cureus.66857

**Published:** 2024-08-14

**Authors:** Haroon Khan, Syed Faqeer Hussain Bokhari

**Affiliations:** 1 Medicine, Naseer Teaching Hospital, Peshawar, PAK; 2 Surgery, King Edward Medical University, Lahore, PAK

**Keywords:** personalized care, digital health interventions, mental health, primary care, depression management, chatbots, artificial intelligence

## Abstract

Depression is a prevalent mental health disorder that significantly impacts primary care settings. This editorial explores the potential of artificial intelligence (AI)-powered chatbots in managing depression within primary care environments. AI chatbots offer innovative solutions to challenges faced by healthcare providers, including limited appointment times, delayed access to specialists, and stigma associated with mental health issues. These digital tools provide continuous support, personalized interactions, and early symptom detection, potentially improving accessibility and outcomes in depression management. The integration of AI chatbots in primary care presents opportunities for round-the-clock patient support, personalized interventions, and the reduction of mental health stigma. However, challenges persist, including concerns about assessment accuracy, data privacy, and integration with existing healthcare systems. Successful implementation requires systematic approaches, stakeholder engagement, and comprehensive training for healthcare providers. Ethical considerations, such as ensuring informed consent, managing algorithmic biases, and maintaining the human element in care, are crucial for responsible deployment. As AI technology evolves, future directions may include enhanced natural language processing, multimodal integration, and AI-augmented clinical decision support. This editorial emphasizes the need for a balanced approach that leverages the potential of AI while acknowledging its limitations and the irreplaceable value of human clinical judgment in depression management within primary care settings.

## Editorial

Introduction

Depression is a pervasive mental health disorder affecting nearly 17 million people in the United States alone, with a significant prevalence in primary care settings [1]. Ranked by the WHO as the fourth leading cause of disability, it shows wide prevalence variability but consistent socio-demographic correlates and adverse effects globally. The 12-month prevalence ranges widely from 0.3% to 23.0% [2]. Given the challenges faced in the primary care management of depression, there is an urgent need for innovative solutions. Artificial intelligence (AI)-powered chatbots have emerged as a promising technological innovation in this field. These advanced digital tools utilize AI and natural language processing to engage with patients, offering potential benefits in depression management, including 24/7 support, initial screenings, psychoeducation, and evidence-based therapeutic interventions.

Depression in primary care

Depression represents a significant and growing burden in primary care settings, with prevalence estimates ranging up to 13% of all primary care visits [3]. The impact on patients is profound, exacerbating existing medical conditions, impairing functional status, and significantly reducing quality of life. Primary care providers face numerous challenges in effectively diagnosing and managing depression, including limited time during appointments, variability in symptom presentation, and stigma associated with mental health disorders. Current standard practices for depression management in primary care typically involve pharmacotherapy and referral to mental health specialists. However, these approaches are often hampered by limitations such as delayed medication effects, long wait times for specialist appointments, and poor integration between primary care and mental health services. The shortage of mental health professionals, particularly in rural and underserved areas, further compounds these challenges.

AI chatbots

AI-powered chatbots are sophisticated software applications designed to simulate human-like conversations through text or voice interactions. In the context of mental health management, these chatbots are programmed with specific knowledge bases and protocols to provide information, support, and basic therapeutic interventions. Several notable examples of AI chatbots used in mental health have emerged in recent years, including Woebot, Wysa, Tess, and Youper [4]. These chatbots demonstrate diverse approaches and applications in mental health, with some focusing on specific therapeutic modalities, like cognitive-behavioral therapy (CBT), while others aim to provide more general emotional support and mood tracking [5].

The integration of AI chatbots into depression management in primary care offers several potential benefits. One of the most significant advantages of AI chatbots is their ability to provide round-the-clock support to patients. Unlike human healthcare providers, chatbots are not constrained by working hours or availability. This continuous accessibility ensures that individuals can receive support at any time, which is particularly crucial for those experiencing acute depressive symptoms or crises outside of regular clinic hours. The immediate availability of support can help prevent the escalation of symptoms and provide timely interventions when needed most. Moreover, AI chatbots can bridge geographical and logistical barriers to care. For patients in rural or underserved areas with limited access to mental health professionals, chatbots can serve as a valuable resource for initial support and guidance. This increased accessibility has the potential to reduce disparities in mental health care delivery and reach populations that might otherwise go untreated.

AI chatbots have the capability to offer personalized interactions based on individual user data and responses. Through machine learning algorithms, these chatbots can analyze patterns in user interactions, symptoms reported, and treatment responses to tailor their approach over time. This personalization can manifest in several ways, including customized psychoeducation, adaptive interventions, personalized goal setting, and tailored recommendations. For instance, chatbots can provide information about depression that is relevant to the individual's specific symptoms and experiences, adjust the type and intensity of therapeutic interventions based on user responses, assist users in setting and tracking personalized goals for managing their depression, and offer suggestions for coping strategies or lifestyle changes that are most likely to benefit the individual user. This level of personalization can enhance the effectiveness of interventions and improve patient engagement with their treatment plan.

The anonymity and privacy offered by AI chatbots can play a crucial role in reducing the stigma associated with seeking help for mental health issues. Many individuals hesitate to discuss their mental health concerns with human providers due to fear of judgment or embarrassment. Chatbots provide a non-judgmental, confidential space for users to explore their feelings and symptoms without the perceived social risks of face-to-face interactions. This reduced stigma can lead to earlier help-seeking behavior and increased willingness to engage with mental health resources. For some individuals, interacting with a chatbot may serve as a stepping stone to seeking professional help, allowing them to become more comfortable discussing their mental health before engaging with human providers.

AI chatbots have significant potential in the early detection of depressive symptoms and timely intervention. Through regular interactions and mood tracking, chatbots can identify patterns or changes in a user's emotional state that might indicate the onset or worsening of depression. This early detection capability is particularly valuable in primary care settings, where depression may often go unnoticed until symptoms become severe. By flagging potential issues early, chatbots can prompt users to seek professional help or alert healthcare providers to follow up with the patient. This proactive approach can lead to earlier interventions, potentially preventing the progression of depressive episodes and improving overall outcomes. Furthermore, AI chatbots can support ongoing monitoring of patients' mental health status between primary care visits. This continuous monitoring can help identify treatment effectiveness, track symptom fluctuations, and alert providers to any concerning changes that require immediate attention.

Challenges and limitations

AI-powered chatbots offer significant potential for managing depression in primary care, yet their effective implementation is fraught with challenges. A primary concern is the accuracy and reliability of AI diagnoses. Unlike human clinicians, AI lacks the ability to interpret nuanced emotional states, non-verbal cues, and context, which are critical in mental health evaluation. This can lead to misdiagnoses or inappropriate recommendations, as current AI systems may struggle with detecting subtle variations in depressive symptoms, distinguishing between similar mental health conditions, and recognizing complex comorbidities. Additionally, AI responses are limited by the data and algorithms on which they are trained, which may not fully capture the diversity of human experiences and cultural contexts.

Data privacy and security are also major concerns, especially given the sensitivity of mental health data. Ensuring secure transmission, protecting against unauthorized access, and navigating international data protection laws are crucial for maintaining patient trust and complying with regulations like the Health Insurance Portability and Accountability Act (HIPAA). Furthermore, integrating AI chatbots into existing healthcare systems poses technical and operational challenges, such as ensuring compatibility with electronic health record systems and managing the additional workload they generate. Crucially, AI chatbots cannot replace human clinical judgment. Clear protocols for when to escalate to human providers, regular review of AI-generated recommendations, and maintaining human oversight are essential for safeguarding patient safety. Ethical considerations, including accountability and the potential for over-reliance on AI, must be addressed to fully realize the benefits of AI in mental health care.

Moreover, the successful integration of AI chatbots in mental health care requires a delicate balance between leveraging technological advancements and maintaining the irreplaceable value of human interaction. While AI can enhance accessibility and efficiency, particularly in resource-limited settings, it is essential to recognize that mental health care is deeply personal and relational. Patients may experience varying degrees of comfort and trust when interacting with AI, and their unique needs may not always align with algorithmic solutions. Therefore, ongoing patient education about the capabilities and limitations of AI chatbots is vital, ensuring that individuals are empowered to seek human support when necessary. Additionally, continuous feedback loops involving both patients and clinicians will be crucial in refining AI tools, making them more adaptive to the complexities of mental health care, and ultimately ensuring that they complement rather than replace human expertise.

Implementation in primary care

Integrating AI chatbots into primary care settings for depression management necessitates a comprehensive, systematic approach that considers various technical, operational, and cultural aspects. The process begins with a thorough assessment of the current technology infrastructure, workflows, and patient care processes within the primary care environment. Engaging all key stakeholders, including primary care providers, mental health specialists, IT staff, and patient representatives, is crucial during the planning and implementation stages. The selection of an appropriate AI chatbot should be guided by the specific needs of the practice, taking into account factors such as integration capabilities, user interface, and evidence supporting its effectiveness. A small-scale pilot testing phase helps identify potential issues and gather valuable feedback from both providers and patients, which is critical for refining the implementation.

Training and educating healthcare providers are essential to the successful deployment of AI chatbots. Providers must be equipped with knowledge of the technical aspects of using the AI chatbot, its capabilities and limitations, and how to interpret and act on AI-generated recommendations. Additionally, they need to understand when to override or supplement AI recommendations with clinical judgment. Communication with patients about the role of AI in their care is also vital. Training should be ongoing, with regular updates provided as the AI system evolves and new evidence about its effectiveness emerges. Collaboration between technology developers and medical professionals plays a key role in creating AI chatbots that meet the practical needs of primary care settings, involving joint development of clinical algorithms, refinement based on clinical feedback, and co-designing user interfaces to align with clinical workflows.

Pilot programs are instrumental in refining the implementation process of AI chatbots in primary care. These programs should be conducted in diverse settings to ensure the generalizability of results and include rigorous evaluation protocols to assess the impact on patient outcomes, provider workload, and healthcare costs. Both quantitative data and qualitative feedback from patients and providers should be collected to capture long-term effects and potential challenges. Case studies from successful implementations can offer valuable insights, detailing the specific context, strategies for overcoming barriers, measurable outcomes, and considerations for long-term sustainability and scalability. By emphasizing collaboration, training, continuous evaluation, and learning from successful case studies, healthcare systems can maximize the benefits of AI chatbots in managing depression in primary care while minimizing risks and disruptions.

Ethical considerations

The integration of AI chatbots into primary care for depression management raises significant ethical considerations. Ensuring informed consent and patient autonomy is crucial, including transparency about the use of AI systems, clear explanations of data usage and protection, and the right to refuse AI-assisted care without compromising access to traditional treatment. Managing biases in AI algorithms is another critical ethical concern. Efforts must be made to ensure that the data used to train AI chatbots is diverse and representative of the population they will serve. Regular audits of AI chatbot performance should be implemented to identify and address any emerging biases or disparities in care recommendations. Balancing technology with the human touch in patient care is essential. While AI chatbots offer numerous benefits, maintaining the human element in mental health care is crucial. The use of AI chatbots should enhance rather than replace the human-to-human therapeutic relationship, and patients should have access to human providers for aspects of care that require genuine empathy and emotional support.

Future directions

As AI technology continues to evolve, the future of AI-powered chatbots in mental health care holds immense potential. Potential advancements include enhanced natural language processing, multimodal AI integrating voice recognition and physiological data, personalized treatment algorithms, and predictive analytics for depressive episodes. The long-term vision for AI in primary care mental health management could include seamless integration into the healthcare ecosystem, continuous monitoring and support for patients, precision mental health with personalized treatment plans, and AI-augmented clinical decision support. AI-powered chatbots represent a promising advancement in mental health care delivery, offering significant potential benefits for depression management in primary care settings. Key advantages include improved accessibility and 24/7 support, personalized interactions and interventions, reduced stigma through anonymity, and early detection and monitoring of depressive symptoms. However, these benefits must be carefully weighed against important challenges, including limitations in AI accuracy and nuanced understanding, data privacy and security concerns, integration difficulties with existing healthcare systems, and ethical considerations around informed consent and algorithmic bias. As we move forward, it is crucial to adopt a balanced approach that recognizes the potential of AI to enhance mental health care while acknowledging its limitations and the irreplaceable value of human clinical judgment and empathy. Ongoing research, collaboration between technology developers and healthcare professionals, and careful consideration of ethical implications will be necessary to realize the full potential of AI chatbots in depression management while ensuring patient safety and well-being. By striking the right balance, we can harness the power of AI to improve mental health outcomes while preserving the compassionate core of medical practice.
